# Supplementary material for: Single neurons in the human medial temporal lobe flexibly shift representations across spatial and memory tasks
Source: bioRxiv. 2023 Feb 23:2023.02.22.529437. Preprint. [Version 2] doi: 10.1101/2023.02.22.529437 (PMC9980106; doi:10.1101/2023.02.22.529437)
Supplement: Supplement 1 [file NIHPP2023.02.22.529437v2-supplement-1.pdf]

bioRxiv preprint doi: https://doi.org/10.1101/2023.02.22.529437; this version posted February 23, 2023. The copyright holder for this preprint (which was not certified by peer review) is the author/funder, who has granted bioRxiv a license to display the preprint in perpetuity. It is made available under aCC-BY 4.0 International license.

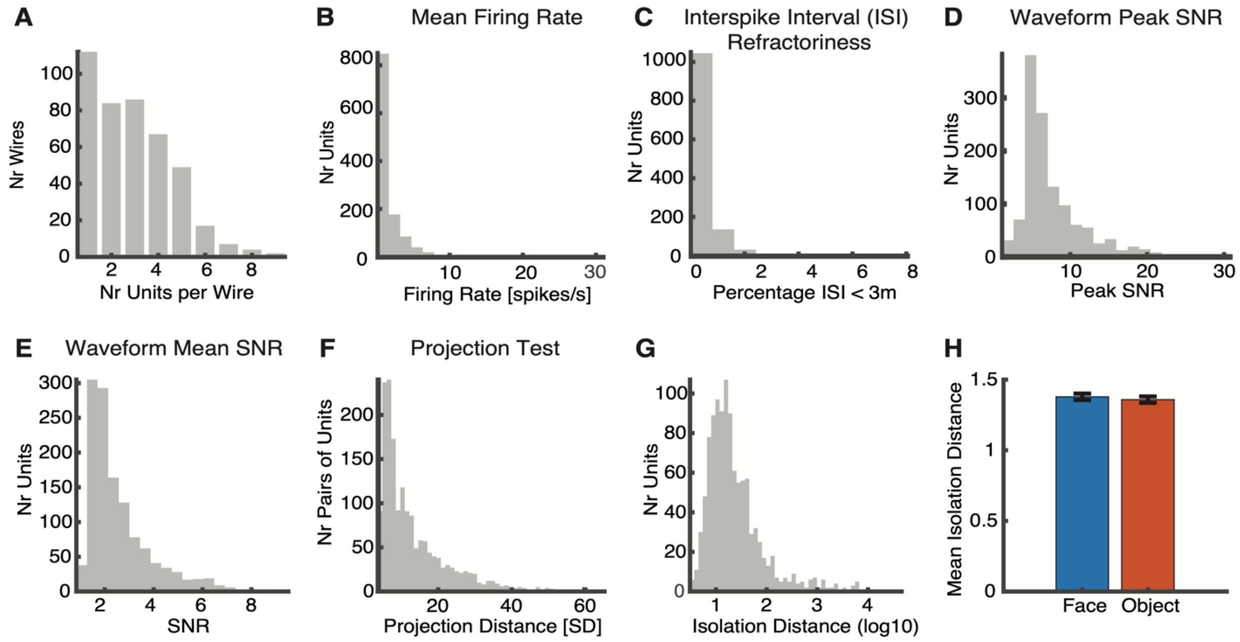

Supplemental Figure 1 - Spike sorting quality measures. Panels A-F reflect spike sorting metrics combined across the face and object versions of the task, including A) the number of units per wire , B) the firing rates, C) the inter-spike intervals, D) the waveform peak signal-to-noise ratio (SNR), E) the waveform mean SNR, F) the projection distance, and G) the isolation distance. G) The mean isolation distance averaged across face and object versions of the paired task sessions.

bioRxiv preprint doi: https://doi.org/10.1101/2023.02.22.529437; this version posted February 23, 2023. The copyright holder for this preprint (which was not certified by peer review) is the author/funder, who has granted bioRxiv a license to display the preprint in perpetuity. It is made available under aCC-BY 4.0 International license.

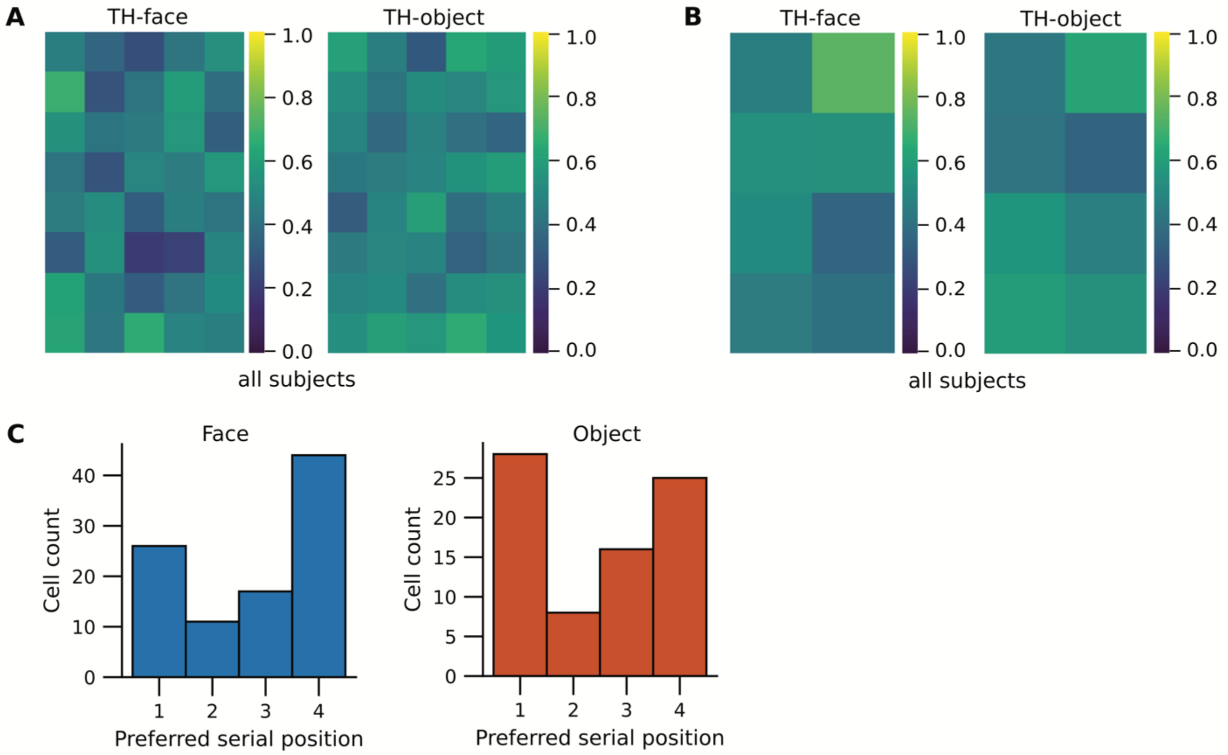

Supplemental Figure 2 - Group-level analyses of the Treasure Hunt task. A) Averaged firing rate maps for all identified place cells, split by face and object versions of the task. B) Averaged firing rate maps from all significant spatial target cells, split by face and object versions of the task. C) The count of preferred positions for all significant serial-position cells, split by face and object versions of the task.
